# Valorization of Livestock Keratin Waste: Application in Agricultural Fields

**DOI:** 10.3390/ijerph19116681

**Published:** 2022-05-30

**Authors:** Huayi Chen, Shuang Gao, Yongtao Li, Hui-Juan Xu, Wenyan Li, Jinjin Wang, Yulong Zhang

**Affiliations:** 1 College of Natural Resources and Environment, Joint Institute for Environmental Research & Education, South China Agricultural University, Guangzhou 510642, China; fengyazi56@163.com (H.C.); gaoshuang0118@163.com (S.G.); yongtao@scau.edu.cn (Y.L.); hjxu@scau.edu.cn (H.-J.X.); lily1984191@scau.edu.cn (W.L.); 2 WENS Foodstuff Group Co., Ltd., Yunfu 527400, China

**Keywords:** biochar, environment, feathers, ionic liquid, keratinases, sustainability, wool

## Abstract

Livestock keratin waste is a rich source of protein. However, the unique structure of livestock keratin waste makes its valorization a great challenge. This paper reviews the main methods for the valorization of livestock keratin waste, which include chemical, biological, and other novel methods, and summarizes the main agricultural applications of keratin-based material. Livestock keratin waste is mainly used as animal feed and fertilizer. However, it has promising potential for biosorbents and in other fields. In the future, researchers should focus on the biological extraction and carbonization methods of processing and keratin-based biosorbents for the soil remediation of farmland.

## 1. Introduction

**Figure 1:**
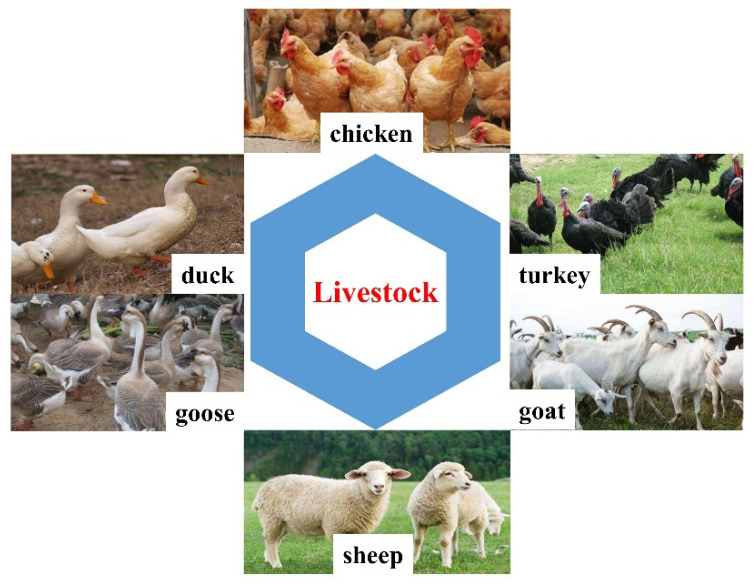
The main livestock producing keratin waste.

**Figure 2:**
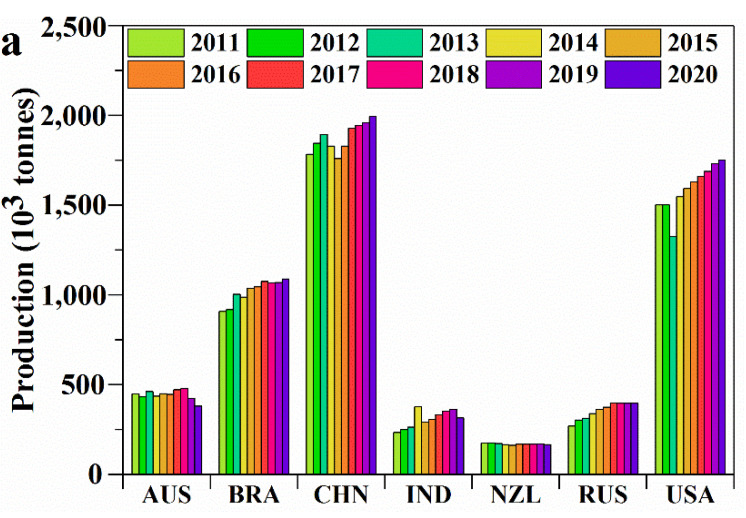

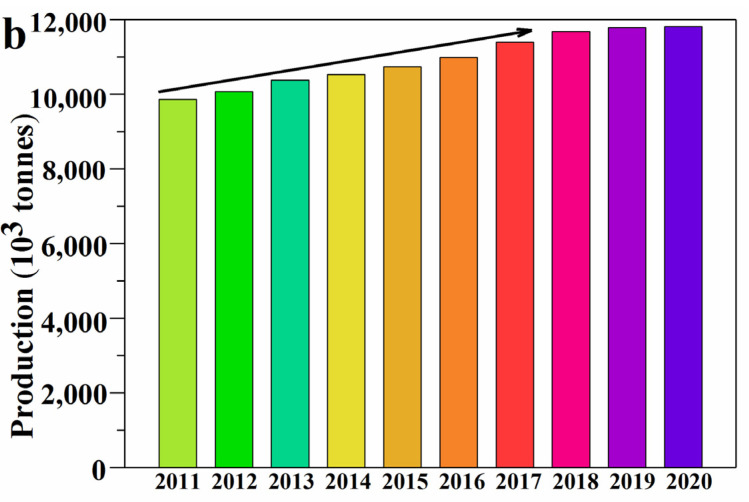
The main livestock keratin waste types (wool and the feathers from goose, guinea fowl, duck, turkey, and chicken) and the production quantity of the main countries (**a**) and in the world (**b**) from 2011−2020. The production quantity of the feathers from goose, guinea fowl, duck, turkey, and chicken is calculated as 7% of body weight.

**Figure 3:**
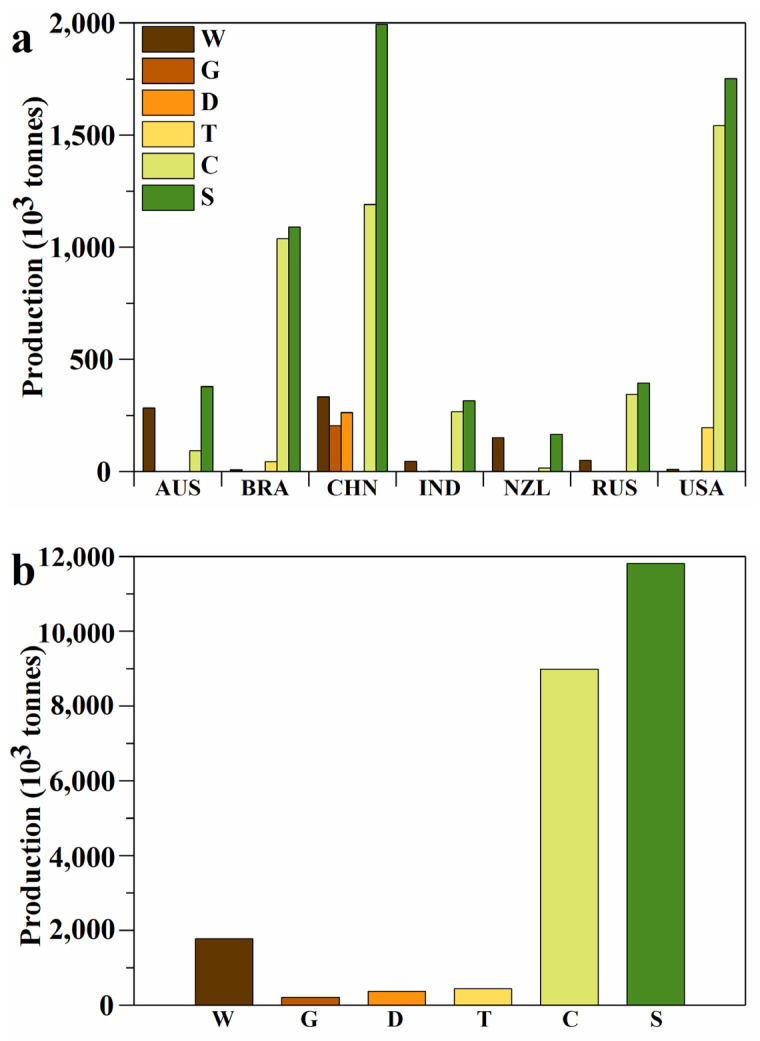
The main livestock keratin waste (wool, and the feather of goose, guinea fowl, duck, turkey, and chicken) production quantity of the main countries (**a**) and the world (**b**) in 2020. W, G, D, T, C, and S represent wool and the feathers of goose and guinea fowl, duck, turkey, and chicken as sum totals. The production quantity of feathers from goose and guinea fowl, duck, turkey, and chicken is calculated as 7% of body weight.

Keratin is an important structural protein found in animal epithelial cells [1]. The main livestock animals producing keratin include chicken, duck, goose, and turkey, as well as goats and sheep (Figure 1). About 95% of wool and up to 90% of feathers are made of keratin [2,3]. With the rapid global development of the intensive breeding industry, large-scale livestock breeding produces millions of tonnes of livestock keratin. The annual production quantity of livestock keratin is increasing year by year. The Food and Agriculture Organization of the United Nations has published data showing that the annual production quantity of keratin, primarily from livestock, has exceeded 10 million tonnes since 2012 (Figure 2). The annual world production of keratin derived mainly from livestock was 11.82 million tonnes in 2020. Figure 3 shows the main keratin production quantities from the main countries in 2020. The top three countries in terms of chicken-feather production are the United States of America, China, and Brazil. The top three countries in terms of wool production are China, Australia, and New Zealand. However, in addition to being processed into wool and down products, most of these resources are discarded or burned. This practice not only wastes valuable protein resources but also causes infections, such as dermatophyte, chlorosis, mycoplasma fowl cholera, and avian influenza, which causes severe health hazards and environmental pollution [4]. Therefore, the efficient processing of livestock keratin waste will not only save resources and reduce carbon emissions but also reduce environmental pollution.

**Figure 4:**
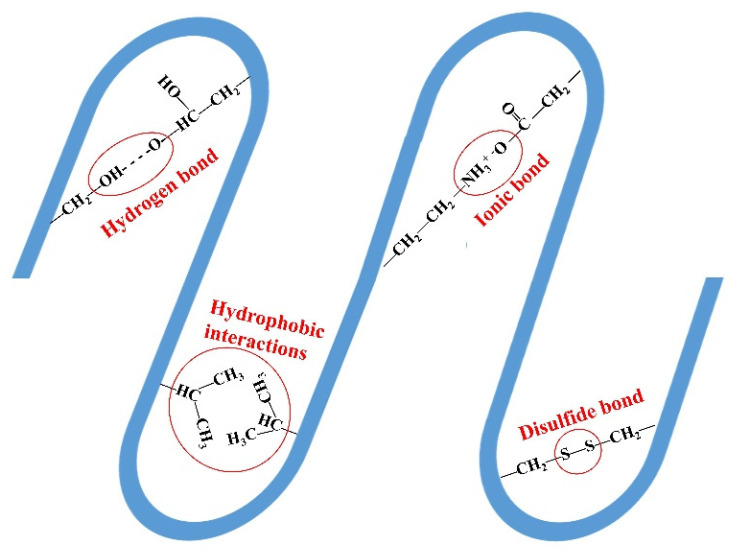
Diagram of the intermolecular and intramolecular bonds of livestock keratin.

However, the unique structure of livestock keratin makes its valorization and application difficult. Keratin has both alpha-helix and beta-fold structures, which protect and maintain its structural stability [5,6]. Alpha-keratin is present in many mammalian tissues and body parts, such as hair, wool, nails, horns, and hooves, while beta-keratin is present in the nails, claws, shells, and beaks of animals [7]. The main chains of the keratin macromolecule can form hydrogen, ionic, and disulfide bonds, as well as hydrophobic interactions (Figure 4) [8]. The formation of disulfide bonds cross-links the polypeptide chain in keratin, making it insoluble in water, salt solutions, diluted acids, and alkali solutions; these disulfide bonds also confer excellent mechanical resistance. Thus, keratin is resistant to the degradation of commonly used proteolytic enzymes [9]. Keratins are grouped into hard (feathers, hair, hoofs, and nails) and soft (skin and callus) types, according to their sulfur contents. Soft keratin contains 2% cysteine, while hard-soft keratin contains up to 10−14% cysteine (hair and wool) or 22% cysteine (horns and nails) [10]. The difficulty in extracting livestock keratin is how to disrupt its disulfide bonds and then dissolve it efficiently. Recently, new and efficient methods to extract livestock keratin for further applications as fiber for spinning, film, and medical materials have been developed. Shavandi et al. reviewed the application of keratin waste in the biomedical industry and provided a theoretical and practical basis for its extraction and application [8]. The literature on livestock keratin waste for agricultural applications is increasing. The application of livestock keratin waste in the agricultural field can reduce carbon emissions and realize sustainable development. Thus, we summarize the main methods for the valorization of livestock keratin and its application in the agricultural industry.

## 2. Methods for the Valorization of Livestock Keratin

### 2.1. Chemical Methods

#### 2.1.1. Acid-Alkali Treatment

Acid-alkali treatment typically immerses livestock keratin waste in a strong acid or alkali solution and then uses hydrolysis, neutralization, and drying to obtain the final product. Common acid or alkali solutions consist of HCl, H_2_SO_4_, Ca(OH)_2_, KOH, and NaOH [11,12,13,14,15,16,17,18,19,20]. The breaking down of peptide and disulfide bonds during the dissolution process creates a discrepancy between the yield and molecular weight of livestock keratin, which needs to be resolved for the acid-alkali treatment. An extended hydrolysis time increases the yield of soluble keratin but will lead to low molecular-weight compounds. In contrast, a short hydrolysis time increases the molecular weight of keratin but decreases the yield. Abou Taleb et al. found that the contents of cysteine, glycine and basic amino acids decreased significantly after treating wool with alkali [21]. Therefore, the appropriate agent and optimal hydrolysis time should be determined according to the intended use of the prepared livestock keratin products after acid-alkali treatment. The most significant disadvantage of acid-alkali treatment is the large amount of alkaline wastewater and waste acid vapor produced during the process, which endangers both equipment and the environment. To save energy and improve the extraction rate, this method has been combined with other methods. The acoustic cavitation-assisted alkaline hydrolysis of wool is an energy-saving process, compared to steam-assisted alkaline hydrolysis, because it can be performed at room temperature and at low pressure [22]. Protein hydrolysate from microwave-alkali treatment contains a significantly higher concentration of amino acids (69.4 mg·g^−1^ of feathers) than the protein hydrolysate of the autoclave-alkali (19.0 mg·g^−1^ of feathers) and conventional heating-alkali (27.8 mg·g^−1^ of feathers) treatments [23].

#### 2.1.2. Oxidation Methods

In 1950, Alexander and Earland reported that wool keratin could be extracted via the oxidation method by treating wool with 2% peracetic acid for 30 h, followed by a mild ammonia (0.2 N) treatment, with a final HCl precipitation step [24]. Peracetic acid, hydrogen peroxide, and formic acid are oxidation agents that are commonly used to extract livestock keratin [17,25,26,27]. These agents can break the disulfide covalent bonds, which results in cysteic acid residues and sulfonate groups; furthermore, this treatment will also form both cysteine monoxide and dioxide [28]. The oxidation method typically extracts more alpha-keratin, which is more soluble than beta-keratin [29]. However, the strong oxidization capacity of these agents can also destroy some amino acids, such as tryptophan, methionine, cysteine, serine, threonine, tyrosine, histidine, and phenylalanine [8].

#### 2.1.3. Reduction Methods

The reduction method employs thiol-containing chemicals to reduce disulfide bonds and is currently widely used. At present, the agents in reduction methods usually consist of denaturants, reducing agents, and surfactants. The denaturant breaks down the hydrogen bonds, which increases the dissolution capacity of livestock keratin. The most commonly used denaturant is urea. The reducing agents consist of 2-mercaptoethanol, cysteine, dithiothreitol, sodium bisulfite, sodium m-bisulfite, sodium metabisulphite, thioglycolic acid, and thiourea [17,18,30,31,32,33,34,35,36,37,38]. However, the reduced keratin will rapidly oxidize and reform disulfide bonds. Consequently, extensive protein aggregation may occur. Thus, surfactants are added to form huge micelles that prevent this situation. The surfactant of choice is typically sodium dodecyl sulfate (SDS). Table 1 shows examples of different reduction methods for the extraction of livestock keratin. Khumalo et al. used a response surface methodology and Box-Behnken design to optimize the extraction process. The results showed that the order of influence on the extraction efficiency of chicken feather keratin is reaction temperature > reaction time > concentration of sodium bisulfite > concentration of SDS [33]. Although these methods are widely used by researchers, they still have many problems and cause instability and secondary pollution.

#### 2.1.4. Ionic Liquid Methods

Ionic liquids (ILs) are currently extensively studied regarding the extraction of keratin. ILs are entirely composed of ions and have melting points below 100 °C. Furthermore, they are regarded as eco-friendly and safe solvents that are non-volatile, non-flammable, easily recyclable, and chemically and thermally stable [1]. In recent years, ILs have often been used to extract keratins from different livestock sources (Table 2). A comparison of different chemical extraction methods by Shavandi et al. shows keratin yields from alkali hydrolysis, reduction, sulfite, oxidation, and IL methods of 63%, 86%, 88%, 93%, and 97%, respectively. These results demonstrate that the keratin yield of ILs was significantly higher than those of the other four methods [17]. Li uses different ILs to dissolve wool, and the results show that 1-allyl-3-methylimidazolium chloride ([AMIM]Cl) IL has a higher solubility for wool keratin than 1-butyl-3-methylimidazolium chloride ([BMIM]Cl) IL [39]. Wang uses a series of synthesized 1-butyl-3-methylimidazolium-based ILs to dissolve wool keratin and reported 1-butyl-3-methylimidazolium dimethyl phosphate ([BMIM][DMP]) as the best solvent. The addition of different co-solvents (e.g., NaHSO_3_, Na_2_SO_3_, SDS, urea, and caprolactam) can shorten the dissolution time [40,41,42]. Additionally, some researchers have used microwave radiation and sonication technology-assisted ionic liquid methods to extract livestock keratin, with excellent results [43,44,45]. Zhang et al. indicated that at least 65% of keratin disulfide bonds should be cleaved to facilitate dissolution in ILs. Moreover, changing the anions and cations can yield dramatically different capabilities of cleaving disulfide bonds, while cation side chains have little effect. Furthermore, dissolution variables, such as time and temperature, and the distribution of the ILs around cysteine have been linked to the ability of ILs to cleave disulfide bonds [46].

### 2.2. Biological Methods

Traditional acid-alkali treatments require considerable energy and destroy several essential amino acids, while oxidation and reduction methods can cause environmental damage. Consequently, biological treatment methods are often applied, including enzymatic hydrolysis and microbial techniques. Biological treatments require less energy consumption and need relatively mild treatment conditions that can avoid the disadvantages of other treatment methods.

#### 2.2.1. Microbial Methods

There are many keratin-hydrolyzing microorganisms that have been isolated from poultry breeding and waste or soil processing, of which the *Bacillus* is the most abundant. Common keratin-degrading microorganisms include *Amycolatopsis* [51], *Bacillus* [52,53,54,55,56,57], *Chryseobacterium* [58,59], *Fervidobacterium* [60,61], *Kocuria* [62], *Lysobacter* [63], *Staphylococcus* [64,65], *Stenotrophomonas* [66,67], *Streptomyces* [68], *Thermoactinomyces* [69,70], *Vibrio* [71,72], and so on. The ability of microorganisms to hydrolyze keratin is determined by amino groups, the mass loss of the keratin substrate, the amino acid profile, substrate alkalization, the release of ammonia/peptides, and the excretion of sulfate or sulfhydryl groups [73]. The pH, temperature, rate of agitation, and sources of carbon, energy, and nitrogen all influence keratin hydrolysis. The complete microbial hydrolysis mechanism of livestock keratin remains unknown, but it is generally considered to occur in two steps: (1) the reduction of disulfide bonds, followed by (2) the cleavage of peptide bonds [74]. Kang et al. further revealed the essential molecular keratinolytic mechanisms from a microbial consortium using shotgun metagenomic sequencing, including amino acid metabolism, disulfide reduction, the urea cycle, and their metabolic cooperation. Meanwhile, more than 90% of genera of the enriched bacterial consortium are affiliated with *Pseudomonas*, *Stenotrophomonas*, and *Chryseobacterium* [75]. These results provide a direction for the practical application of complex communities and contribute to the industrial popularization of microbial methods.

#### 2.2.2. Enzymatic Hydrolysis Methods

Enzymatic hydrolysis methods are similar to microbial methods. The key difference is that microbial methods use the activity of the whole microorganism to hydrolyze keratin, while enzymatic hydrolysis uses the cell-free extracts of keratinases produced by specific microorganisms. Due to its unique structure (highly cross-linked disulfide bonds, hydrophobic interactions, and hydrogen bonds), livestock keratin cannot be hydrolyzed by common proteases, such as pepsin, trypsin, and papain [76]. Therefore, the hydrolysis of livestock keratin requires keratin-specific enzymes known as keratinases. Keratinases are proteases that are produced by certain microorganisms, and that can hydrolyze keratin to release soluble proteins, peptides, and amino acids. It is reported that keratinases are produced by some microorganisms, such as *Amycolatopsis* [77], *Arthrobacter* [78], *Aspergillus* [79], *Bacillus* [2,56,80,81,82,83,84,85], *Lysobacter* [63], *Micrococcaceae* [86], *Ochrobactrum* [87], *Paenibacillus* [88], *Stenotrophomonas* [67,89,90], *Streptomyces* [91,92], *Vibrio* [71], and so on. These keratinases are active over a broad range of pH values (5−12) and temperatures (20 °C−55 °C). Sulfitolysis is likely a major step in the hydrolysis of livestock keratin, which precedes the action of all keratinases. Their efficiency can be evaluated by measuring the enzymic activity, the concentration of both the released thiol groups and soluble proteins, and the incurred weight loss [8]. Since the cost of keratinase production is high, several researchers have increased the yield and stability of keratinases by altering the genome of keratinolytic organisms via physical and chemical mutagenesis or recombinant DNA technologies [80,82,90,93,94,95,96]. Keratinases have become increasingly popular, and many studies have reviewed their discovery, production, classification, structure, hydrolysis function, and industrial application [9,97,98,99,100]. Biological techniques are environmentally friendly methods to hydrolyze keratin, but they require more time than other methods. If the large-scale and rapid industrial production of keratinases and microorganisms can be realized, biological methods are expected to become a widely used method for the valorization of livestock keratin.

### 2.3. Other Methods

Additionally, some novel methods have been used by researchers, such as steam flash explosions [101,102], microwave-assisted extraction [103,104], and deep eutectic solvents [105,106]. Deep eutectic solvents have been used instead of ILs because they exhibit low vapor pressure and non-flammability [106]. These methods aim to improve the extraction yield of livestock keratin. However, they also have limitations, such as a high cost or a complex mechanism. Thus, continued research into and the improvement of livestock keratin extraction methods are required. Furthermore, more and more studies have pyrolyzed livestock keratin waste into carbon materials instead of extracting keratin from it [107,108,109,110,111,112,113,114]. However, livestock keratin waste will produce toxic gases during carbonization, such as CO, HCN, H_2_S, and SO_2_ [115,116]. The problem of toxic gas emissions must be solved in the industrial production of keratin-based carbon materials to avoid secondary pollution. It is necessary to compare the performance, energy consumption, carbon budget, and costs of the carbonization method and hydrolysis method. Meanwhile, livestock keratin waste is often mixed with other waste (excrement, eggshell, and so on) found on the farm. To extract livestock keratin using traditional methods, livestock keratin waste must first be separated. If livestock waste can be carbonized without sorting to yield high-efficiency carbon materials, the step of separating livestock waste can be removed, thereby reducing the costs and realizing the high valorization of livestock keratin waste.

## 3. Application of Livestock Keratin Waste in Agriculture

### 3.1. Animal Feed

Livestock keratin waste is rich in amino acids (Table 3) and can be used as animal feed. Previous studies have shown that some animals have an excellent ability to digest livestock keratin powder. The effective and full application of livestock keratin waste is of great significance to produce keratin powder, which alleviates the lack of protein in conventional feed. Chicken feather waste that has been treated with Ca(OH)_2_ for 3 h at 150 °C (about 95% of which was dissolved) results in livestock keratin hydrolysate, which is rich in amino acids and polypeptides. This could be used as an animal-feed additive. However, this livestock keratin hydrolysate is deficient in arginine, histidine, lysine, methionine, and threonine. These particular amino acids are required for monogastric animals; therefore, it is recommended for use as a ruminant supplement [13]. Feathers treated with *Kocuria rosea* to obtain fermented feather powder reach a pepsin digestibility of 88%, as well as contents of protein, lysine, histidine, and methionine of 71%, 3.46%, 0.94%, and 0.69%, respectively. Furthermore, the available amino acid content measured in vivo is higher than that in commercial feather powder. This indicates that the feather powder obtained after treatment with *Kocuria rosea* can be used as a protein-rich feed [62]. In aquaculture, many results show that it is feasible to partially replace other protein sources with livestock keratin. However, the effective substitution level varies depending on the quality and processing methods of the feather powder, as well as the type, size, and breeding conditions of the test fish [117,118]. The immune response in both the intestine and liver of hybrid tilapia that were fed with hydrolyzed feather meal (instead of soybean or cottonseed meal) indicates this to be an excellent alternative protein source, which induces less stress in the host [119]. Furthermore, the digestibility of feather keratin when treated with keratinase (0.985) is similar to that of both casein (0.994) and soy protein (0.995) and exceeds that of untreated feather meal (0.578) [71]. Moreover, in recent years, most feed application scenarios used hydrolyzed feather meal produced via biological methods [54,57].

### 3.2. Fertilizer

Livestock keratin waste contains a large number of nutrients, such as carbon, nitrogen, and sulfur (Table 4). These elements have good potential to promote plant growth and improve soil quality. Therefore, livestock keratin waste can be used as fertilizer. However, compared with other waste, the mineralization rate of livestock keratin waste in the soil is slow [122], so it cannot be directly used as fertilizer in the soil. The methods of preparing livestock keratin waste as fertilizer can mainly be divided into two categories. The first category involves the direct application of prepared livestock keratin hydrolysate to the soil as fertilizer. It has been reported that the number of germinated seeds and the dry weight of wheat supplemented with 0.1% *w*/*w* wool hydrolysates produced using acoustic-assisted alkaline hydrolysis was 11% and 84% higher than that of control [22]. The chicken feather hydrolysate degraded by *Chryseobacterium* sp. RBT has been used as a general and foliar fertilizer for banana plants. The chlorophyll content of the thus-fertilized banana leaves increased from 0.89 mg·g^−1^ to 1.43 mg·g^−1^, the protein content of the banana fruits increased from 15.1 mg·g^−1^ to 16 mg·g^−1^, and the amino acid content increased from 2 mg·g^−1^ to 2.96 mg·g^−1^, compared to controls. These results show that hydrolyzed feathers can be used as a cheap and efficient fertilizer, thus supporting the material’s continued research and popularization [58]. Livestock keratin hydrolysate has also been used to fertilize other crops [51,88]. In addition, livestock keratin hydrolysate contains a large number of amino acids, so it is often used as an amino acid fertilizer. Feather-based fertilizers containing 15% (AminoPrim) and 20% (AminoHort) amino acids were applied in the field. The results showed that AminoPrim (dose: 1.00 L·ha^−1^) and AminoHort (dose: 1.25 L·ha^−1^) can increase the grain yield of winter wheat by 5.4% and 11.0%, and increase the quality of the grain, such as its protein and nutrient contents [123]. Chicken-feather keratin hydrolysate treated with H_2_SO_4_ and KOH is used to prepare amino-acid-chelated Zn and Fe fertilizers, ethylenediaminetetraacetic acid-chelated Zn and Fe fertilizers, and zinc sulfate and iron sulfate fertilizers. These fertilizers can be used as foliar fertilizers for rice, increasing plant growth and chlorophyll content [15].

Although most studies have focused on the use of livestock keratin hydrolysate as a fast-release fertilizer, the use of livestock keratin for the production of slow-release fertilizers has also been studied. Synthesized nano-keratin, produced via the reduction method, is coated with urea to supply nitrogen, which is then encapsulated in a coconut spathe to achieve the slow release of nutrients [128]. Double-coated controlled-release urea fertilizer is prepared using chicken-feather keratin and corn straw as coating materials, which improves the total nitrogen use efficiency and increases the soil water retention capability [129]. A multifunctional eco-friendly fertilizer prepared with a keratin-based superabsorbent as an outer coating material can be used for slow-release urea and soil remediation [130]. Keratin-based hydrogel is one of the slow-release fertilizers and has excellent properties. Su et al. prepared novel chicken-feather keratin grafted poly(potassium acrylate)/polyvinyl alcohol hydrogels by graft copolymerization, and the results showed that these hydrogels had good water retention properties and nitrogen and phosphorus adsorption properties [19]. Meanwhile, the maximum release values of nitrogen and phosphorus from the hydrogel were 69.46% nitrogen and 65.23% phosphorus in the field’s soil within 30days, which values are best fitted by the Ritger–Peppas equation [16]. The use of livestock keratin to produce fertilizers improves the economic value of livestock keratin waste, protects the environment, reduces pollution, and achieves the sustainable development of resources. As a new and green fertilizer, the market prospects of livestock keratin fertilizer will likely increase. Meanwhile, biochar produced from livestock keratin waste can be used as a carbon-based fertilizer. Biochar has many functions, such as carbon sequestration, increasing soil nutrients, improving soil quality, and so on [131,132]. However, little research has been conducted on the use of livestock keratin-based biochar as a fertilizer in soils. Researchers may pay more attention to this avenue in the future.

### 3.3. Biosorbents

Keratin molecular chains contain many functional groups, such as amino (R-NH_2_), carboxyl (R-COOH), hydroxyl (R-OH), and sulfhydryl groups (R-SH); therefore, keratin is a potential adsorption material. Livestock keratin waste is often used as a biosorbent to remove both heavy metals and organic pollutants in solutions. Livestock keratin waste can be used as a biosorbent in two major ways. The first is to use livestock keratin waste directly as a biosorbent (Table 5). Chicken feathers were used to absorb acid Blue 80 dye. When the initial concentration of acid Blue 80 dye is 5 × 10^−5^ mol·L^−1^, chicken feathers (at a dose of 2.5 mg·L^−1^) can adsorb about 80% acid Blue 80 dye at 50 °C [133]. Raw chicken feathers can absorb 4.31 mg·g^−1^ Zn^2+^ at 30 °C and pH 5, and 7.84 mg·g^−1^ Cu^2+^ at 30 °C and pH 3 [124,134]. Our previous studies show that the Cd^2+^ and Pb^2+^ adsorption capacities of raw chicken feathers were 4.32 mg·g^−1^ and 18.42 mg·g^−1^ at 25 °C and pH 5, respectively [110]. The results reported by Nikiforova et al. indicate that the Cd^2+^, Cu^2+^, Ni^2+^, and Zn^2+^ adsorption capacities of wool fibers are all less than 1 mg·g^−1^ [135].

Most of the results of the direct application of livestock keratin waste as biosorbents are not so successful. Although livestock keratin waste has many functional groups, these functional groups may not be the active adsorption sites of pollutants or they may not be exposed at the surface, resulting in low adsorption capacities. Therefore, many researchers use the hydrolysates of livestock keratin waste as biosorbents, including keratin nanofibers, keratin sponges, keratin hydrogel, and so on (Table 6). Sun et al. prepared a chicken-feather biosorbent using a sodium dodecylbenzene sulfonate solution pretreatment and dissolution with a [BMIM]Cl IL that could adsorb 63.5–87.7% Cr^6+^ at concentrations from 2 mg·L^−1^ to 80 mg·L^−1^ [48]. The wool hydrolyzed by a reduction method was prepared into keratin nanofibers by electrospinning and was then used to adsorb Cu^2+^, Ni^2+^, and Co^2+^ [30,31]. Wool keratin hydrolyzed by the reduction method can be prepared as keratin nanofibers by electrospinning. The maximum adsorption capacity of keratin nanofibers for methylene blue is 167 mg·g^−1^ [139]. Wool keratin prepared using the reduction method is used to prepare regenerated keratin sponge, which has a high adsorption capacity for both liquid paraffin and soybean oil of over 30 g·g^−1^ [38]. In addition, more and more researchers have blended keratin with other materials to produce composites. Wool keratin hydrolyzed by NaOH has been used to prepare keratin/polyamide 6-blend nanofibers, which have a high Cr^6+^ adsorption capacity of over 55.9 mg·g^−1^ [11]. The AzureA and Methyl Orange maximum adsorption capacities of wool keratin/hydrotalcite hybrid sponges were reported as 0.20 mmol·g^−1^ and 0.04 mmol·g^−1^ at room temperature and neutral pH, respectively [140]. The Pb^2+^ adsorption capacity of polyacrylic acid/wool keratin (product from reduction method) hydrogel can reach 234.60 mg·g^−1^ at 25 °C and at a pH of 4. Besides this, using traditional methods to extract livestock keratin will produce some by-products (residues) that are often wasted, thus reducing the utilization rate of livestock keratin waste. Gao et al. prepared a Cr^6+^ adsorbent from the residue of chicken-feather keratin extracted by the reduction method, that reached a maximum Cr^6+^ adsorption capacity of 21.35 mg·g^−1^ [32]. It presents a new idea for the application of livestock keratin. Briefly, the by-products of incomplete livestock keratin extractions contain macromolecules with various groups of livestock keratin that can also be used as biosorbents. Additionally, more and more studies have converted livestock keratin waste into carbon-based sorbents (Table 7). However, our previous results showed that the unmodified chicken feather-based biochar has a poor adsorption capacity for Cd^2+^ and Pb^2+^, while the adsorption capacities of biochar when modified by phosphoric acid and potassium hydroxide were greatly improved [110,111]. Therefore, although livestock keratin waste has the potential to produce biochar due to its abundant functional groups, how to produce keratin-based biochar with high adsorption capacities is still a major challenge.

### 3.4. Other Aspects

Research on livestock keratin in different agricultural fields is constantly being published. Slaughterhouse waste, such as bones, blood, intestines, and feathers with high protein and lipid contents can be converted into biogas by anaerobic digestion [141]. The production of biogas from livestock keratin waste offers an important application prospect. Dried feathers have 0.05 m^3^·kg^−1^ wet-weight methane potential and 0.2 m^3^·kg^−1^ VS_added_ [142], and the yield is typically improved by pre-treatment, which is a common method for producing biogas from livestock keratin waste. Methane production from dry feathers after pretreatment with recombinant *Bacillus megaterium* of 0.35 Nm^3^·kg^−1^ has been achieved. This corresponded to 80% of the theoretical value of proteins [52], while the methane yield from feathers pretreated with *Bacillus* sp. C4 was 124% higher than that of untreated feathers [55]. Ca(OH)_2_, thermal, and enzymatic pretreatments have also been used to produce biogas from chicken feathers and wool textile residues [14,143].

Additionally, livestock keratin-based materials have also been applied to soils to improve the soil quality and environment. Feather hydrolysate prepared by *Streptomyces sampsonii* GS1322 is rich in amino acids and proteins and is applied to barren agricultural land soil. This results in an increased wheat germination rate (1.25 times) and plant height (1.18 times) 15 days after sowing and 90 days after planting, respectively, compared to control plants. Furthermore, after 90 days, the total number of soil microorganisms, ammonifying bacteria, and phosphate-solubilizing bacteria increased by 3.19, 2.17, and 1.18 times, respectively, while the total number of pathogenic fungi was only 32% of that in the control. Thus, feather hydrolysate can be used as a low-cost soil amendment to fertilize poor farmland [68]. Feather hydrolysate, obtained using a mixed culture of *Thermoactinomyces* strains, has been applied to native park soil and anthropogenically affected soil, where it has improved seed germination and ryegrass growth. The results show that the total number of soil microorganisms and ammonifying bacteria increased, and the feather hydrolysate exhibited excellent resistance to various bacteria. This research indicates that feather hydrolysate can be used as an organic conditioner and biocontrol agent to repair polluted soil and accelerate ryegrass growth [69]. Several chitin- and keratin-rich organic amendments are used to improve soil quality. The results show that these amendments can improve the bacterial and fungal microbial soil communities and reduce *Rhizoctonia solani* disease symptoms in sugar beet plants, which indicates that chitin- and keratin-rich organic amendments can increase plant and soil resilience and/or disease suppression [144].

## 4. Conclusions

The annual production of livestock keratin waste is enormous and is continuously increasing; therefore, it is necessary to study its valorization and application. Here, we review the valorization methods of livestock keratin. (1) The traditional methods of livestock keratin extraction (acid-alkali treatment, oxidation, and reduction) have limitations and may possibly cause secondary pollution. Consequently, these traditional methods are not suitable for the future extraction of livestock keratin. (2) ILs have become a research hotspot as a new type of livestock keratin solvent. ILs have great potential due to advantages such as a high extraction rate, green operation, and low pollutant formation. Thus, research on and designing suitable ILs to extract livestock keratin have become an important research direction. (3) Biological methods have gradually become the main focus of research due to their high extraction rate, low pollution, and low cost. The main difficulty in popularizing biological methods is that industrial production is not feasible, and most reported research is still at the laboratory stage. (4) The carbonization of livestock keratin wastes into carbon materials is a new method for its valorization, but it is necessary to first solve the problem of tail-gas emissions. (5) Therefore, applying these methods to industrial production is a problem that needs to be resolved through the combined effort of the researchers and industry.

Here, we summarize the main agricultural applications of keratin-based material. (1) The practical application of livestock keratin in agriculture mainly focuses on its use as animal feed and fertilizer. However, the types and amounts of the keratin-derived amino acids produced varied significantly, depending on the types of livestock keratin waste and extraction methods. This impacts the palatability of the animal feed. Thus, attention should be paid to palatability testing and cooperative feeding alongside other feeds. Research into livestock keratin-based fertilizers should not be limited to the input of nutrients but should also address its effects on soil properties (physical and chemical), microorganisms, and pollution. (2) Currently, many studies report the use of livestock keratin waste as a biosorbent due to its specific functional groups, which can complex or adsorb pollutants (organics and heavy metals). In addition to increasing the application of livestock keratin in water treatment, future research should also investigate the use of environmentally friendly products for the remediation of polluted soil and to improve soil quality. (3) Applications should be developed according to different livestock keratin waste situations and agricultural contexts, with different methods to treat livestock keratin waste and when making different products, so that the high valorization of livestock keratin waste can be realized.

## Figures and Tables

**Table 1:** Extraction of livestock keratin by different reduction methods.

Keratin Waste	Reducing Agents	Denaturant	Surfactant	Condition	Refs.
wool	2-mercaptoethanol	urea	SDS	60 °C, 12 h	[17]
wool	sodium metabisulphite	urea	SDS	30–100 °C, 0.25–0.75 h	[38]
wool	sodium metabisulphite	urea	/	65 °C, 2 h	[30]
wool	cysteine	urea	/	85 °C, 12 h	[36]
feathers	cysteine	urea	/	75 °C, 9.5 h	[35]
chicken feathers	thiourea	urea	SDS	50 °C, 12 h	[37]
chicken feathers	sodium bisulfite	urea	SDS	80 °C, 4 h	[32]
chicken feathers	2-mercaptoethanol	/	/	50 °C, 12 h	[18]
chicken feathers	sodium bisulfite	/	/	50 °C, 12 h	[18]
chicken feathers	sodium m-bisulfite	/	/	50 °C, 12 h	[18]
chicken feathers	dithiothreitol	/	/	50 °C, 12 h	[18]

**Table 2:** Extraction of livestock keratin using different ionic liquid methods.

Keratin Waste	Ionic Liquid	Conditions				Refs.
		Ratio (IL/Keratin Waste)	Time (h)	T (°C)	Other	
duck feathers	[BMIM]Cl	20:1	1	90	10 wt.% Na_2_SO_3_	[40]
turkey feathers	[BMIM]Cl, [AMIM]Cl, [choline][thioglycolate]	20:1	10	130	/	[47]
chicken feathers	[HOEMIm][NTf2]	40:1	4	80	1:1 NaHSO_3_/feather	[42]
chicken feathers	[BMIM]Cl	77:23	48	100	/	[48]
wool	[BMIM]Cl	10:1	2	130	/	[17]
wool	[BMIM][DMP]	20:1	10.5	120	/	[41]
wool	[BMIM]Cl	40:1	10	130	/	[49]
wool	[BMIM]Cl	6:1	0.5	120, 150, 180	/	[50]

**Table 3:** The contents of amino acids in different livestock keratin waste (mol%).

Amino Acids	Untreated Feathers [120]	Untreated Wool [121]
ALA	8.40	5.76
ARG	1.70	7.30
ASP	6.70	6.43
CYS	7.60	5.65
GLU	9.70	12.23
GLY	16.20	9.44
HIS	0.30	0.80
ILE	4.30	3.45
LEU	8.30	8.08
LYS	1.80	3.01
MET	/	0.59
PHE	4.30	2.93
PRO	18.80	7.08
SER	7.20	10.96
THR	0.80	6.38
TYR	1.60	3.51
VAL	2.00	6.38

**Table 4:** The element contents in the different forms of livestock keratin waste (wt %).

Element	Chicken Feathers [110]	Chicken Feathers [124]	Chicken Feathers [125]	Wool [126]	Pig Hair [127]
C	44.18	47.65	47.40	46.52	44.2
N	13.69	9.98	15.10	18.98	14.50
H	7.28	7.49	7.20	6.28	6.00
S	2.30	1.44	2.90	3.15	3.40

**Table 5:** Adsorption of different pollutants by raw livestock keratin waste.

Livestock Keratin Waste	Removal Rates	Refs.
hen feathers	4.00 × 10^−4^ mol·g^−1^ (Brilliant Blue FCF) at 30 °C and pH 2	[136]
hen feathers	1.75 × 10^−5^ mol·g^−1^ (erythrosine dye) at 30 °C and pH 3	[137]
hen feathers	1.20 × 10^−4^ mol·g^−1^ (tartrazine) at 30 °C and pH 2	[138]
chicken feathers	about 80% acid Blue 80 dye at 30 °C, C_0_ = 5 × 10^−5^ mol·L^−1^, dose = 2.5 mg·L^−1^	[133]
chicken feathers	4.31 mg·g^−1^ (Zn^2+^) at 30 °C and pH 5	[124]
chicken feathers	4.32 mg·g^−1^ (Cd^2+^) and 18.42 mg·g^−1^ (Pb^2+^) at 25 °C and pH 5	[110]
chicken feathers	7.84 mg·g^−1^ (Cu^2+^) at 30 °C and pH 3	[134]
wool	0.46 mg·g^−1^ (Zn^2+^), 0.48 mg·g^−1^ (Ni^2+^), 4.85 mg·g^−1^ (Cu^2+^), and 0.73 mg·g^−1^ (Cd^2+^) at 25 °C and pH 6	[135]

**Table 6:** Adsorption of different pollutants by biosorbents prepared from livestock keratin waste hydrolysate.

Biosorbents	Adsorption Capazcities	Refs.
Wool keratin nanofiber prepared by electrospinning	167.00 mg·g^−1^ (methylene blue) at 20 °C and pH 6	[139]
Wool keratin/hydrotalcites hybrid sponge	0.20 mmol·g^−1^ (AzureA) and 0.04 mmol·g^−1^ (Methyl Orange) at room temperature and neutral pH	[140]
Pigeon-feather keratin sponge	over 30.00 g·g^−1^ (liquid paraffin and soybean oil)	[38]
Chicken-feather particles	adsorb 63.50–87.70% Cr^6+^ at concentrations from 2 mg·L^−1^ to 80 mg·L^−1^	[48]
Wool keratin nanofiber prepared by electrospinning	30.00 mg·g^−1^ (Cu^2+^) at 20 °C and pH 6	[31]
Wool keratin nanofiber prepared by electrospinning	3.26 mol·g^−1^ (Co^2+^), 3.66 mol·g^−1^ (Ni^2+^) and 4.85 mol·g^−1^ (Cu^2+^) at 25 °C and pH 6	[30]
Polyamide 6/wool keratin blend nanofiber	55.90 mg·g^−1^ (Cr^6+^) at pH 6	[11]
Feather keratin/dialdehyde cellulose nanocrystals hybrid sponge	517.00 mg·g^−1^ (Cd^2+^) and 767.00 mg·g^−1^ (Pb^2+^) at room temperature and pH 5.5	[35]
Wool keratin sponge	270.27 mg·g^−1^ (Cr^6+^)	[36]
Chicken-feather keratin/polyacrylic acid hydrogel	234.60 mg·g^−1^ (Pb^2+^) at 25 °C and pH 4	[37]
By-product of hydrolyzing chicken feathers	21.35 mg·g^−1^ (Cr^6+^) at 30 °C and pH 6	[32]

**Table 7:** Adsorption of different pollutants by livestock keratin waste-based carbon materials.

Raw Materials	Modify Agents	Adsorption Capacities	Refs.
Chicken feathers	/	5.68 mg·g^−1^ (Cd^2+^) and 40.93 mg·g^−1^ (Pb^2+^) at 25 °C and pH 5	[110]
Chicken feathers	H_3_PO_4_	7.84 mg·g^−1^ (Cd^2+^) and 55.42 mg·g^−1^ (Pb^2+^) at 25 °C and pH 5	[110]
Feathers and *Acorus calamus Linn.*	H_3_PO_4_	56.64 mg·g^−1^ (Cr^6+^) at room temperature and pH 3	[109]
Chicken feathers and eggshell	FeCl_3_·6H_2_O and FeCl_2_·4H_2_O	35.70 mg·g^−1^ (Ni^2+^), 49.12 mg·g^−1^ (Zn^2+^), 58.79 mg·g^−1^ (Cu^2+^), 66.21 mg·g^−1^ (Cd^2+^), and 68.00 mg·g^−1^ (Pb^2+^) at 25 °C and pH 5.5	[108]
Chicken feathers	KOH	62.14 mg·g^−1^ (Cd^2+^) and 143.00 mg·g^−1^ (Pb^2+^) at 25 °C and pH 5	[111]
Chicken feathers	KOH	103.57 mg·g^−1^ (amoxicillin)	[112]
Chicken feathers	KOH	388.33 mg·g^−1^ (tetracycline) at 30 °C	[107]
Cow hair	KOH	1477.00 mg·g^−1^ (direct blue dye) at 25 °C	[114]
Animal hair	H_3_PO_4_	0.89 mmol·g^−1^ (norfloxacin) and 0.41 mmol·g^−1^ (acetaminophen) at 25 °C	[113]

## Data Availability

Not applicable.
